# Barriers and Facilitators of Re-Employment among Senior Workers: Prospective Cohort Study

**DOI:** 10.3390/ijerph191811536

**Published:** 2022-09-13

**Authors:** Kristina Thomassen, Emil Sundstrup, Jonas Vinstrup, Karina Glies Vincents Seeberg, Lars Louis Andersen

**Affiliations:** 1 National Research Centre for the Working Environment, 2100 Copenhagen, Denmark; 2 Department of Health Science and Technology, Aalborg University, 9220 Aalborg, Denmark

**Keywords:** re-employment, return to work, unemployment, unemployment characteristics, seniors, occupational, worker

## Abstract

Re-entering the labour market after a period of unemployment can be challenging for seniors. This study investigates personal as well as circumstantial barriers and facilitators of re-employment. Unemployed seniors in Denmark (≥50 years, n = 1636) from the first wave (mid-2018) of the SeniorWorkingLife study were prospectively followed until March 2020 in national registers on labour market participation. Using weighted logistic-regression-modelled odds ratios (ORs), we estimated the association between personal and circumstantial factors at baseline and re-employment during follow-up. During follow-up, 28% re-entered paid employment. The desire to have a job (reference: not having the desire to have a job) increased the likelihood of re-employment (OR 2.35, 95% CI 1.14–4.85). Contrastingly, a higher age (60–63 vs. 50–54 years; OR 0.36, 95% CI 0.16–0.79) and poor health (OR 0.32, 95% CI 0.16–0.61) decreased the likelihood of re-employment. Sex, education and belief that age constitutes a barrier to re-employment were not associated with the likelihood of re-employment. Unemployed seniors desiring to have a job are more likely to get a job. However, a higher age and poor health are important barriers that should be taken into account, e.g., by ensuring employment opportunities for these groups in society.

## 1. Background

Growing populations approaching the age of retirement have led to political reforms with the aim to meet the shortage in the labour force [1]. Recently, there has been a focus on how to retain working seniors, but perhaps a more pressing challenge is how unemployed seniors who desire to work are effectively re-employed. Not only is it valuable for society to re-employ seniors, but being a part of the labour market also contributes to seniors’ everyday structure and is associated with feeling useful and their overall mental and physical well-being, whereas being outside the labour market may have both negative personal and financial consequences [2,3,4,5,6].

To address this issue, it is salient to improve the chances for seniors to return to work after a period of unemployment, as there are currently more barriers than opportunities for seniors’ chances of re-employment. For example, studies exploring employers’ perceptions of seniors show that negative conceptions of hiring senior workers, age discrimination, and negative conceptions about seniors’ ability to work still act as barriers to re-employment [7,8]. Furthermore, even though seniors are generally considered to be loyal, reliable, and experienced, they have more difficulties re-entering the labour market compared to younger applicants [9,10]. Likewise, long-term unemployment (>1 year) also decreases the likelihood of re-employment, as employers often consider long-term unemployment as a sign of incompetency and lack of motivation [11,12].

Lastly, poor health among senior workers makes it even more difficult to maintain a job [13]. The bidirectional relationship between unemployment and poor health has been well established in the literature; it is illustrated by a higher prevalence of disability, illness and mortality rates among unemployed individuals [14,15]. Therefore, identifying several of the barriers and prerequisites for the successful re-employment of senior workers in a prospective manner is crucial.

In this study, we investigated barriers and facilitators of re-employment among unemployed seniors, which is an essential step toward developing effective societal guidelines aimed at re-employment, thereby retaining senior workers longer in the labour market. We hypothesized that poor health, shorter education and higher age were barriers for re-employment and that wanting to have a job was a facilitator.

## 2. Methods

### 2.1. Study Design and Population

We used data from the first wave of the SeniorWorkingLife study combined with a prospective follow-up in national registers on labour market participation. 

The SeniorWorkingLife is a project investigating push and stay mechanisms for labour market participation among older workers (≥50 years) in Denmark. Push refers to mechanisms that increase the risk of a premature exit from the labour marker, e.g., due to age discrimination, poor health and a poor working environment. Stay refers to mechanisms prolonging working life, e.g., due to a good working and social environment and attractive working conditions [16]. In SeniorWorkingLife, 30,000 participants aged 50 years or older, including 7000 unemployed individuals, were randomly drawn by Statistics Denmark and invited to participate with a personal questionnaire link via e-Boks (online digital mailbox linked to the Danish social security number). In the present study, we included data on unemployed senior workers from the first round of the SeniorWorkingLife study, with a maximal age of 63 years (i.e., they would not receive state pension during follow-up) and who confirmed on the questionnaire that they were still unemployed; this resulted in a final sample size of 1636 unemployed seniors.

All data were linked by use of the personal identification number, a unique identifier assigned to all Danish residents at birth or after immigration [16,17,18].

### 2.2. Predictors

From the SeniorWorkingLife study, the questions regarding facilitators for re-employment included: (1) “*Would you like to have a job?*” and (2) “*Do you believe that your age is a barrier to re-employment*?”.

The first question was answered on a dichotomized yes/no scale, whereas the latter questions were answered on a 5-point Likert scale; i.e., *“To a very high degree”*, *“To a high degree”*, *“To some degree”*, *“To a slight degree”*, *and “Not at all”.*

We used data from two Danish nationwide registries: the Danish Civil Registration System [17], with information on age and sex, and from Statistics Denmark [18], with information on the level of education. 

Based on education, respondents were stratified into groups of either short or long education. Short education is typically vocational education of around 1–2 years in duration and long education is typically 3 to 5 years in duration.

### 2.3. Outcome

The outcome variable “paid employment” in 2020 was assessed from a national register on labour market affiliation, administered by Statistics Denmark. Paid employment was defined as those who, in March 2020, fulfilled the following three criteria: First, the person had been in paid employment for at least 20 h per week for at least half of 2019. Second, the person was employed at least 20 h per week during March 2020. Third, the person did not receive benefits from “flexible jobs” (a job offer on special terms for people with permanently reduced work ability), “light jobs” (work on special terms with a wage subsidy offered to people on a disability pension), sickness absence or maternity/paternity leave during the first quarter of 2020. Seniors who fulfilled all three criteria were defined as “re-employed”. 

### 2.4. Statistical Analysis

The SurveyFreq procedure (SAS version 9.4, Cary, North Carolina) was used to produce estimates of prevalence, whereas the SurveyLogistic procedure was used to produce odds ratios (ORs); both included 95% confidence intervals (95% CI), and analyses were mutually adjusted. (i.e., they were controlled for each other). 

Model-assisted weights were used to produce representative estimates, accounting for different sizes and response percentages of subgroups. This procedure ensured that the estimates were representatives. Model-assisted weights were based on information from high-quality national registers at Statistics Denmark and controlled for education, sex and age.

## 3. Results

Table 1 shows the demographics of the study population. The majority of the study population had no education or a shorter education (64%) and 77% reported their health status as good or very good. Overall, the majority would like to have a job (80%). Lastly, more than half of the study population believe (to a “very high” or “high degree”) that their age constitutes a barrier for re-employment (53%). At follow-up, 28% were re-employed.

Table 2 shows the association between barriers and facilitators predicting re-employment. Individuals who desired to have a job experienced increased odds of re-employment during follow-up (OR 2.35, 95% CI (1.14–4.85)) compared to individuals not wanting a job. Individuals with poor self-perceived health were less likely to be re-employed during follow-up (OR 0.32, 95% CI (0.16–0.61)) compared to individuals with good self-perceived health. Likewise, age was associated with re-employment, where those in the oldest age group (60–63 years) were significantly less likely to return to the labour market compared to their younger counterparts; 60–63 vs. 50–54 years (OR 0.36, 95% CI (0.16–0.79)). However, believing that age constitutes a barrier was not associated with re-employment. Lastly, education and sex were not significant predictors of re-employment.

## 4. Discussion

This study investigated which factors—personal as well as circumstantial—are important for returning to the labour market among unemployed seniors. The main facilitator was the desire to be re-employed and the main barriers were higher age and poor health. 

In general, the vast majority stated that they would like to have a job. This is in line with previous research showing that the majority of unemployed seniors are flexible and willing to compromise by, e.g., accepting a different job in order to return to the labour market [19]. Not surprisingly, our results show that individuals having the desire to return to the labour market were significantly more likely to be re-employed. 

Interestingly, although more than half of the study population believe that their age constitutes a potent barrier for re-employment, the prospective study design did not show any such associations. In contrast, actual age did constitute a barrier for re-employment, with the likelihood of re-entering paid employment decreasing markedly with increasing age. Several reasons may underlie this discrepancy. For example, higher age may affect seniors’ motivation to continue to work, and seniors not believing that they will return to the labour market could also be an indication that they are expecting to retire shortly [1]. However, in line with previous studies, a higher age affects seniors’ chances of re-employment as negative attitudes towards older job applicants based on stereotypes, such as age discrimination, still pose a problem, which highlights the difficulties for senior workers of being re-employed once unemployed [8,20]. To meet this challenge, measures should be taken in order to equalize seniors’ chances within the labour market, utilizing a targeted approach. For example, in Denmark, a new law was enacted in 2022 prohibiting employers from asking for the job applicants’ age when applying for a job. This may be an important first step to prevent age discrimination among job applicants.

Due to demographic changes across Europe, there are strong political interests in prolonging and maintaining the labour force and, therefore, there is a large body of literature investigating factors of importance for a prolonged working life. Across various job groups, the possibility of more senior days, followed by greater flexibility, are factors that seem important for prolonging seniors’ working life [21]. Furthermore, a prospective study showed that a good psychosocial work environment appears to facilitate working beyond the state pension age, including for those with physically active work [22]. However, differences also seem to exist between different occupational groups: a higher proportion of seniors with mainly sedentary work tasks would stay longer if there were possibilities for more senior days, longer vacations and more flexible working, whereas seniors with physically demanding work would stay longer if the work was less physically demanding [21]. Although this study deals with unemployed seniors, this fits well with a study investigating factors conditioning retirement decisions [23]. Factors such as not being able to do the required work and poor physical health were more prominent among seniors with a short education compared to seniors with a long education. 

The ability to continue working until an old age is very much affected by health. Especially among skilled or low-skilled workers with physically demanding jobs, the combination of high physical work demands and health is a strong predictor of poor work ability [24]. Concurrently, the risk of poor health increases with age, challenging senior workers to cope with the job demands, especially when the physical and mental work demands are high [25,26]. In the current study, poor health was a strong barrier to returning to the labour market. Although it cannot be excluded that seniors with already poor health are clustered among the unemployed in this study, these findings support the large body of literature reporting an association between health and labour market affiliation, where poor mental and physical health is one of the biggest reasons for not working [27,28,29,30,31].

The majority of the study population had a shorter education. The social gradient in health and socioeconomic positions, where less-educated individuals, often with high physical work demands, have a lower work ability than individuals with longer education, is one of the key determinants of extending working lives among employed seniors [4,32]. However, in contrast to common beliefs, shorter education was not a barrier for returning to paid employment. Thus, although employed senior workers with longer educations often work until a higher age, once unemployed, it is equally difficult for those with longer and shorter educations to re-enter the labour market. 

Summarily, as unemployment among seniors is a growing issue with increasing societal consequences, this study highlights important individual and circumstantial barriers to re-employment. Although it may seem intuitive that an increased motivation for being active in the labour market often increases the odds of that happening, the fact that actual and perceived barriers to re-employment do not always align is problematic. Likely influenced by stereotypical assumptions about senior workers, this warrants future research on what constitutes actual barriers and opportunities among senior workers.

### Strengths and Limitations 

The primary strength of the study is the prospective design with a register follow-up, as it provides clarity of the temporal sequence. Furthermore, using model-assisted weights based on national registers ensures that the sample is representative of Danish unemployed seniors. Although generalizable to senior workers in Denmark and the Scandinavian countries, the generalizability of this study to other countries is limited. 

## 5. Conclusions

Unemployed seniors who desire to work were more likely to re-enter the labour market. Once unemployed, it is equally difficult for seniors with longer and shorter educations to re-enter the labour market. However, a higher age and poor health significantly decrease the likelihood of being re-employed. Such knowledge is relevant for the development of societal guidelines aimed at the re-employment of senior workers, thereby retaining workers longer in the labour market.

## Figures and Tables

**Table 1:** Characteristics of the study population.

	N	Percent %
**Gender**		
Female	890	54.5
Male	746	45.5
**Age, years**		
50–54 years	477	29.2
55–59 years	634	38.8
60–63 years	525	32.0
**Education**		
Shorter education	1052	64.3
Longer education	584	35.7
**Self-reported health status**		
Good health (good to excellent)	1265	77.3
Poor health (fair to poor)	371	22.7
**Re-employed at follow-up**		
Yes	464	28.4
No	1172	71.6
**Do you think your age is a barrier to re-enter the labour market?**		
From not at all to some degree	696	47.0
From a high degree to a very high degree	940	53.0
**Would you like to have a job?**		
From not at all to some degree	368	20.0
From a high degree to a very high degree	1268	80.0

**Table 2:** Prevalence and odds ratio of barriers and facilitators of re-employment. Significant associations are marked in bold.

Factors for Returning to the Labour Market	Odds Ratios (95 % CI *)
**Would You Like to Have a Job?**	
From not at all to some degree	1
From a high degree to a very high degree	**2.35 (1.14–4.85)**
**Self-reported health**	
Good (good to excellent)	1
Poor (fair to poor)	**0.32 (0.16–0.61)**
**Age**	
50–54 years	1
55–59 years	1.01 (0.57–1.79)
60–63 years	**0.36 (0.16–0.79)**
**Do you believe your age is a barrier to re-enter the labour market?**	
From not at all to some degree	1
From a high degree to a very high degree	1.27 (0.75–2.18)
**Education**	
Longer education	1
Shorter education	1.06 (0.64–1.77)
**Gender**	
Men	1
Women	1.12 (0.69–1.81)

* Analyses were mutually adjusted for all variables in this table.

## Data Availability

The authors encourage collaboration and use of the data by other researchers. Researchers interested in using the data for scientific purposes should contact the project leader Prof. Lars L. Andersen, lla@nfa.dk.
